# The Role of Traditional Chinese Medicine in Metabolic Reprogramming, Tumor microenvironment, and Macrophage Regulation: Mechanisms and Advances in Preclinical Studies

**DOI:** 10.7150/jca.126434

**Published:** 2026-04-22

**Authors:** AnAn Yu, Donglai Yang, Hongyu Chen

**Affiliations:** 1 Hangzhou TCM Hospital of Zhejiang Chinese Medical University (Hangzhou Hospital of Traditional Chinese Medicine), No. 453 Tiyuchang Road, Xihu District, Hangzhou 310007, China; 2 Zhejiang Hospital, No. 12 Linyin Road, Xihu District, Hangzhou 310007, China.

**Keywords:** Traditional Chinese Medicine (TCM), Metabolic Reprogramming, Macrophage Polarization

## Abstract

**Background:**

Cancer metabolism reprogramming is a hallmark of tumorigenesis, playing a critical role in tumor progression and therapeutic resistance. Macrophages in the Tumor microenvironment (TME) play a crucial role in tumor progression and immune system evasion. Traditional Chinese Medicine (TCM) has shown potential in influencing cancer metabolism and immune functions, offering new therapeutic possibilities.

**Objective:**

This review explores the intersection of TCM, metabolic reprogramming, and macrophage polarization in cancer, focusing on their molecular mechanisms and therapeutic implications. The study further seeks to summarize preclinical evidence supporting TCM's role in modulating tumor metabolism and immune microenvironments, offering a holistic approach to cancer therapy.

**Methods:**

In this study, we systematically reviewed the latest research progress in the last five years on the role of traditional Chinese medicine in regulating tumor metabolic reprogramming, TME and macrophage polarization. A comprehensive literature reviews the metabolic shifts in cancer cells, including glycolysis, oxidative phosphorylation, and lipid metabolism, and how TCM compounds restore metabolic balance. Studies on macrophage polarization (M1/M2) and their regulatory roles in cancer immunity, as influenced by TCM, were analyzed. Preclinical research highlighting TCM's dual impact on tumor metabolism and the TME was also evaluated.

**Results:**

TCM compounds demonstrate the ability to target key metabolic pathways, such as glycolysis and lipid metabolism, restoring metabolic homeostasis in cancer cells. Additionally, TCM has been shown to regulate macrophage polarization within the TME, enhancing M1 macrophage-mediated anti-tumor immunity and suppressing M2 macrophage-mediated tumor promotion. Preclinical studies further support TCM's capacity to modulate the TME and improve therapeutic outcomes when used in combination with conventional cancer therapies.

**Conclusions:**

TCM integrates metabolic reprogramming and immunomodulation to provide a comprehensive treatment strategy for cancer. However, translating these findings into clinical practice remains challenging. Future research should focus on validating the exact mechanisms of TCM in tumor metabolism and immunity through clinical trials and investigating the synergistic effects of TCM with modern cancer therapies, such as immunotherapy and targeted therapies. This may facilitate the use of TCM as an adjuvant therapy to improve the efficacy of existing cancer treatments.

## 1. Introduction

Cancer remains a leading cause of death worldwide. According to statistics, an estimated 20 million people will be diagnosed with cancer by 2022, and the number of diagnoses is expected to increase by 77 percent by 2050 to 35 million 1. And despite significant advances in treatment modalities, including surgery, chemotherapy, and immunotherapy, therapeutic resistance and relapse continue to challenge effective management. Increasing evidence indicates that tumor progression and therapy resistance are closely linked to coordinated alterations in cellular metabolism and immune regulation within the TME 2. A hallmark feature of cancer is metabolic reprogramming, wherein tumor cells alter their metabolic pathways to support uncontrolled growth, survival, and metastasis. Importantly, tumor metabolic reprogramming not only fuels malignant cell growth but also reshapes the functional state of immune cells, particularly tumor-associated macrophages, thereby reinforcing immune suppression within the TME 3. Recent studies have identified tumor-associated macrophages (TAMs) as key players in shaping the TME (TME), modulating both cancer progression and the immune response 4-6. Macrophages can exist in two major phenotypic states: M1 macrophages, which are anti-tumoral, and M2 macrophages, which promote tumor progression and immune evasion. The polarization of macrophages within the TME can significantly influence tumor progression and the response to treatment 7-9.

Traditional Chinese Medicine (TCM) has a long history of use in cancer therapy and is gaining increasing attention due to its potential to modulate multiple biological pathways, including metabolic reprogramming and immune modulation 10-12. TCM offers a holistic approach to cancer treatment by balancing the body's internal environment, improving systemic immunity, and influencing cancer cell metabolism 13-15. This multi-target and system-level regulatory capacity positions TCM as a potential immunometabolic modulator rather than a single-target cytotoxic intervention 16. Many compounds present in TCM, such as flavonoids, alkaloids, and polysaccharides, have been found to inhibit cancer cell growth by modulating key metabolic pathways such as glycolysis, oxidative phosphorylation, and lipid metabolism 17-19.

Emerging evidence suggests that TCM may also impact the macrophage polarization within the TME, shifting the balance from an M2 (tumor-promoting 4 phenotype to an M1 (tumor-suppressing) phenotype, thereby enhancing anti-tumor immunity and potentially overcoming immune evasion mechanisms 20-22. Such macrophage reprogramming is increasingly recognized as a key determinant of therapeutic responsiveness to chemotherapy and immunotherapy. The combination of metabolic reprogramming and immune modulation through TCM could provide a novel therapeutic strategy for cancer treatment 23-25.

**Figure 1:**
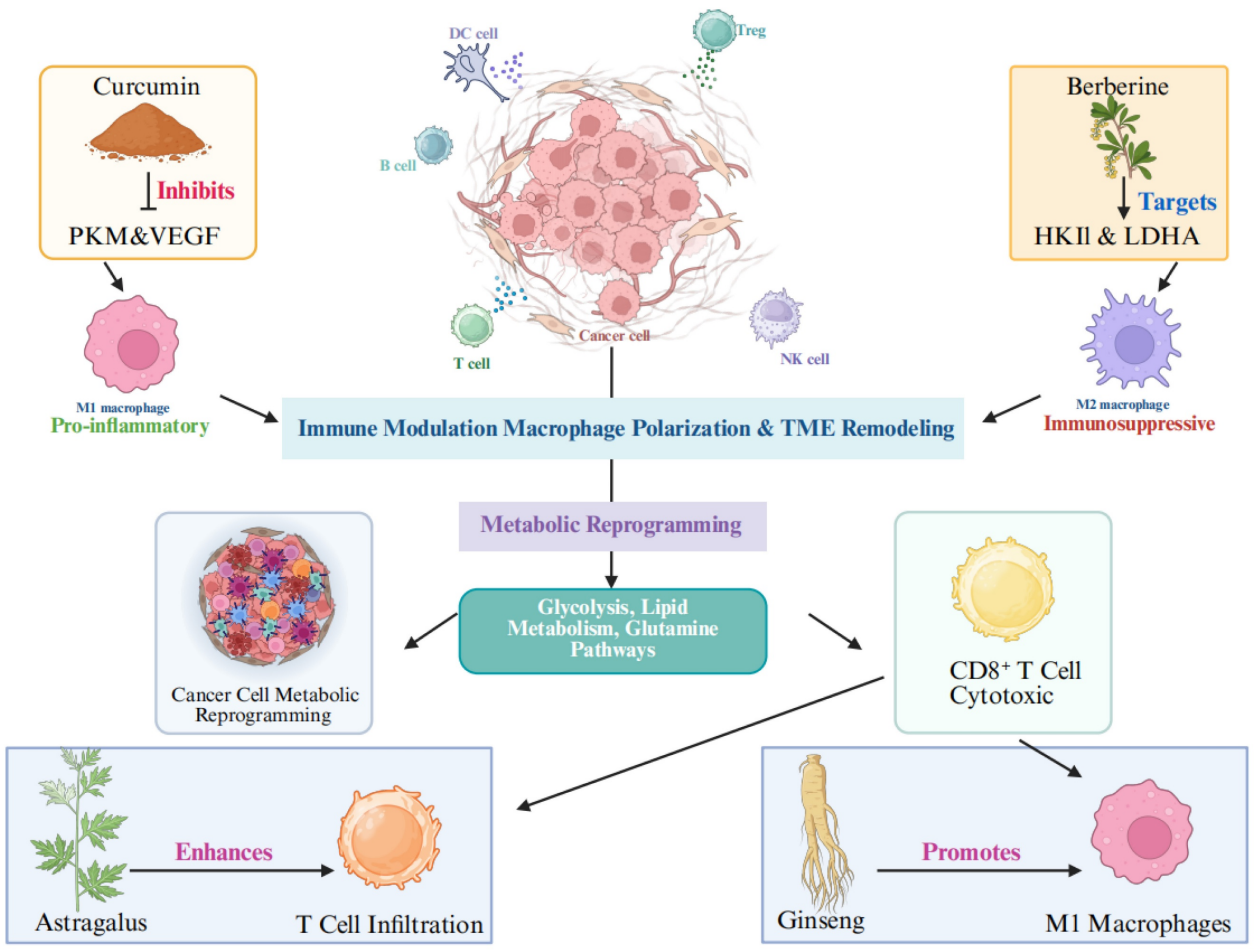
Mechanism of tumor suppression via immune-metabolic modulation by TCM.

As shown in Figure 1, this review aims to explore the intersection of TCM, metabolic reprogramming, and macrophage modulation in cancer therapy, with an emphasis on the molecular mechanisms through which TCM compounds regulate both cancer metabolism and macrophage polarization. We will also discuss preclinical and clinical studies that demonstrate the potential of TCM to enhance the efficacy of conventional cancer therapies by modulating the TME, thereby offering a promising adjunctive approach to cancer treatment.

## 2. Cancer Metabolic Reprogramming

### 2.1. Overview of metabolic reprogramming in cancer

Metabolic reprogramming enables tumor cells to adapt to the hostile microenvironment, sustain rapid proliferation, and evade cell death 26-27. This feature was formally recognized as one of the “Hallmarks of Cancer”. In healthy cells, glucose metabolism primarily relies on oxidative phosphorylation (OXPHOS) in mitochondria. However, even under aerobic conditions, cancer cells favor glycolysis for adenosine triphosphate (ATP) production, a phenomenon known as the Warburg effect. This metabolic shift not only provides rapid ATP but also supplies intermediates for biosynthesis, essential for cellular proliferation 28-29.

**Figure 2:**
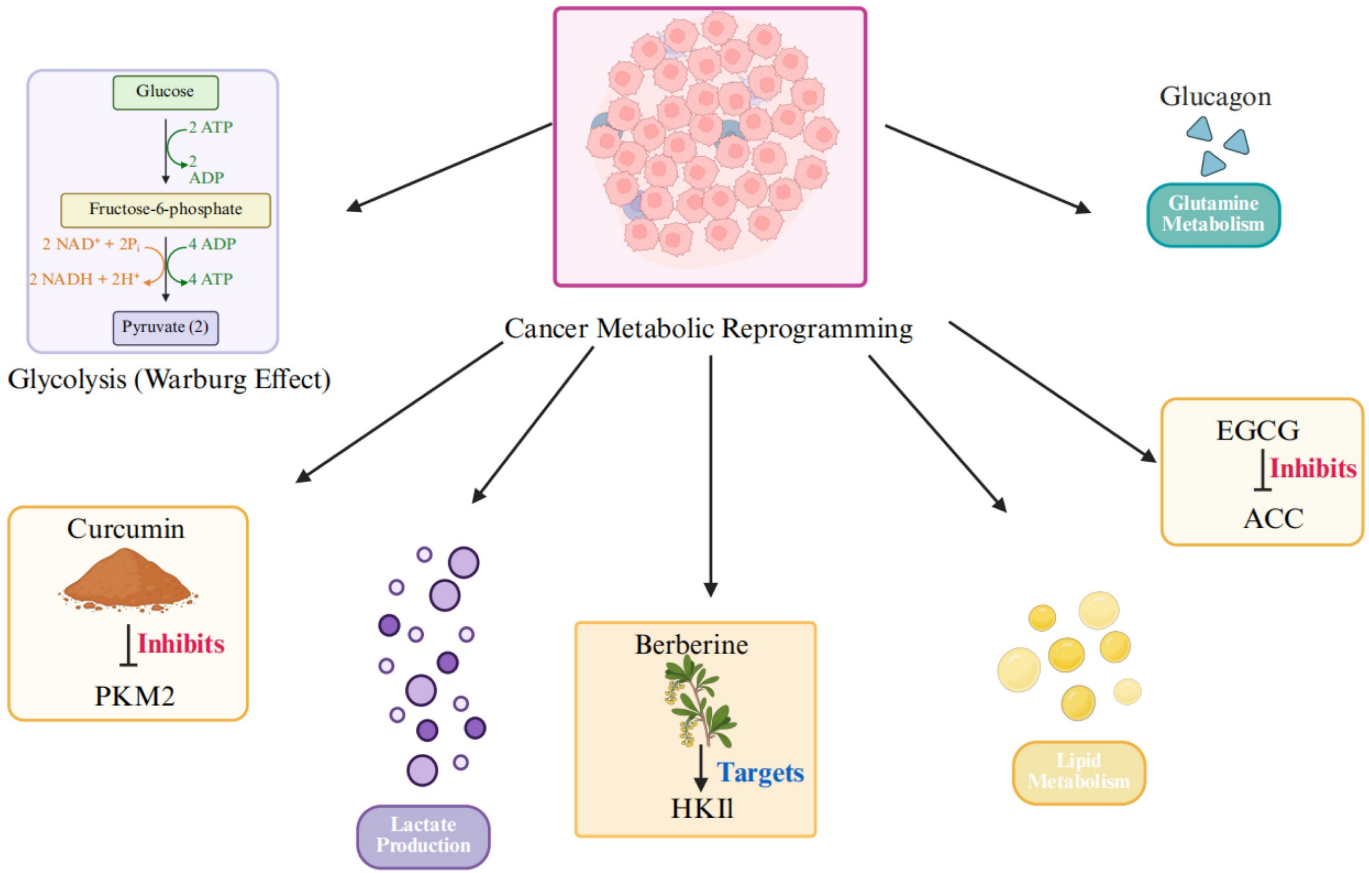
TCM target key metabolic reprogramming pathways in cancer.

As shown in Figure 2, beyond the Warburg effect, cancer cells exhibit reprogramming in several other metabolic pathways, including enhanced lipid metabolism, glutamine dependency, and lactate-driven immune suppression. These pathways collectively form the foundation for tumor proliferation, survival, and metastasis 30-32.

### 2.2. Major Reprogrammed Metabolic Pathways in Cancer

#### 2.2.1. Permission to reuse and Copyright

The Warburg effect is characterized by the preference of cancer cells for glycolysis over OXPHOS, even in the presence of oxygen. Glycolysis allows cancer cells to quickly generate metabolic intermediates required for nucleotide, protein, and lipid synthesis 33-34. Key enzymes in this pathway, such as Hexokinase II (HKII), Pyruvate Kinase M2 (PKM2), and Lactate Dehydrogenase A (LDHA), are often overexpressed in cancer cells and regulated by oncogenes like MYC and HIF-1α 35-36. Targeting glycolysis has shown significant therapeutic potential 37-38. For instance, Berberine, a bioactive compound from Coptis chinensis, activates AMPK and downregulates HKII expression, reducing glycolytic flux and lactate production, ultimately suppressing tumor growth 39. Similarly, Curcumin inhibits PKM2 activity, preventing the conversion of pyruvate to lactate, which significantly impairs colorectal cancer cell proliferation 40.

#### 2.2.2. Lactate Metabolism and Tumor

Microenvironment lactate, once considered a waste product of glycolysis, has emerged as a critical regulator of the TME. Accumulated lactate acidifies the TME, which suppresses the activity of effector T cells and NK cells, while promoting the expansion of regulatory T cells (Tregs), thereby facilitating immune evasion 41-44. Baicalin, a flavonoid from Scutellaria baicalensis, has been shown to reduce lactate production, reprogramming the TME and enhancing T cell-mediated anti-tumor immunity 45-47.

#### 2.2.3. Lipid Metabolism

Lipid Metabolism is essential for cancer cells to support membrane biosynthesis, energy storage, and signaling. Enhanced expression of Fatty Acid Synthase (FAS) and Acetyl-CoA Carboxylase (ACC) is a hallmark of reprogrammed lipid metabolism in various cancer cells, including breast and prostate cancer 48. Epigallocatechin gallate (EGCG), a green tea-derived polyphenol, inhibits FAS activity, leading to reduced lipid synthesis and subsequent inhibition of tumor growth and invasion 49,50.

#### 2.2.4. Glutamine Metabolism

Many cancer cells exhibit glutamine addiction, relying on glutamine as a source of carbon and nitrogen for nucleotide, amino acid, and lipid biosynthesis. Through the action of Glutaminase (GLS1), glutamine is converted into glutamate, which enters the TCA cycle to support anabolic metabolism. Astragalus polysaccharides, extracted from Astragalus membranaceus, downregulate GLS1 expression, reducing glutamine dependency and suppressing tumor growth 51,52.

### 2.3. Metabolic Reprogramming and Therapy Resistance

#### 2.3.1. Chemotherapy Resistance

Enhanced glycolysis increases ATP production, enabling cancer cells to effectively pump out chemotherapeutic agents via ATP-dependent efflux pumps, reducing drug sensitivity. Overexpression of HKII and LDHA has been closely linked to resistance to drugs like cisplatin and doxorubicin 53.

#### 2.3.2. Immunotherapy Resistance

Lactate accumulation in the TME suppresses the activity of effector immune cells, diminishing the efficacy of immune checkpoint inhibitors such as anti-PD-1 and anti-CTLA-4 antibodies. Baicalin, by reducing lactate production, has been shown to enhance the efficacy of these therapies in preclinical models 54.

### 2.4. The Roles of Traditional Chinese Medicine in Targeting Metabolic Reprogramming

#### 2.4.1. Restoring Metabolic Balance

Berberine activates AMPK and inhibits mTOR signaling, effectively disrupting the metabolic demands of cancer cells and restoring energy homeostasis. Tanshinone IIA, derived from *Salvia miltiorrhiza*, modulates the PPARγ pathway, reducing lipid accumulation and significantly inhibiting breast cancer cell migration and invasion 55-56.

#### 2.4.2. Improving the Metabolic Environment

Baicalin reduces lactate levels in the TME, alleviating the acidic microenvironment and enhancing T cell function 54. Curcumin targets the PDK1/LDHA axis, decreasing lactate production and reversing immune suppression in the TME 57.

#### 2.4.3. Improving the Metabolic Environment

EGCG inhibits FASN, sensitizing cancer cells to chemotherapeutic agents such as paclitaxel 58. Astragalus polysaccharides have demonstrated synergistic effects with PD-1 inhibitors, significantly enhancing anti-tumor efficacy in mouse models 59-60.

## 3. The TME: An Expanded Background

### 3.1. Overview of the TME

The TME (TME) is a dynamic and complex ecosystem surrounding cancer cells, comprising various stromal cells, immune cells, vascular endothelial cells, fibroblasts, extracellular matrix (ECM) components, and signaling molecules. Unlike normal tissue environments, the TME plays a dual role in tumor development: it can either suppress tumor progression in its early stages or promote cancer growth, metastasis, and resistance to therapy as the tumor evolves 61-62.

The TME is highly heterogeneous and constantly evolving, influenced by interactions between cancer cells and their surrounding components. These interactions shape cancer progression, immune evasion, angiogenesis, and resistance to conventional and targeted therapies. By modifying the TME, cancer cells create an environment conducive to their survival and proliferation. Importantly, the TME is not only a passive bystander but an active participant in the cancer progression process, making it an important therapeutic target in modern oncology 63-65.

### 3.2. Key Components of the TME

#### 3.2.1. Immune Cells and Immune Modulation

The TME hosts a variety of immune cells, including tumor-associated macrophages (TAMs), myeloid-derived suppressor cells (MDSCs), regulatory T cells (Tregs), dendritic cells (DCs), natural killer (NK) cells, and cytotoxic CD8+ T cells. These immune cells are critical in determining the fate of tumor growth and metastasis, as they mediate immune surveillance and immune evasion mechanisms 66-67.

#### 3.2.2. Tumor-Associated Macrophages (TAMs)

TAMs are highly plastic and can polarize into either the M1 phenotype (pro-inflammatory and anti-tumorigenic) or the M2 phenotype (anti-inflammatory and pro-tumorigenic). TAMs in the TME are predominantly polarized towards the M2 phenotype, secreting cytokines like IL-10 and TGF-β, which suppress immune responses and promote tumor progression. High M2 macrophage infiltration is associated with poor prognosis in many cancers, including breast, lung, and ovarian cancer 68-70.

#### 3.2.3. Regulatory T Cells (Tregs)

Tregs are recruited to the TME through chemokines like CCL22 and CCL28. Once in the TME, Tregs suppress cytotoxic T cell function and secrete immunosuppressive cytokines, such as IL-10 and TGF-β, creating an immunosuppressive microenvironment. This immune evasion mechanism allows tumors to grow unchecked and resist immune-based therapies 71-72.

#### 3.2.4. Effector Cells (CD8+ T Cells and NK Cells)

Despite their tumor-killing potential, effector immune cells often become dysfunctional in the TME due to metabolic competition, hypoxia, and inhibitory signaling pathways. For instance, lactate accumulation from the Warburg effect directly inhibits CD8+ T cell function and prevents NK cell activation 73-74.

#### 3.2.5. Vasculature and Angiogenesis

Tumor angiogenesis is one of the hallmarks of cancer and plays a critical role in providing nutrients and oxygen to rapidly proliferating cancer cells. The process is driven by vascular endothelial growth factor (VEGF), which stimulates the formation of new blood vessels. However, these tumor-associated blood vessels are often aberrant, leaky, and poorly organized, leading to hypoxia within the TME. Hypoxia further promotes tumor progression by stabilizing hypoxia-inducible factor 1-alpha (HIF-1α), which enhances glycolysis, angiogenesis, and metastasis. Abnormal vasculature also limits the delivery of chemotherapeutic agents and immune cells to the tumor core, posing significant challenges to effective treatment 75,76.

#### 3.2.6. Cancer-Associated Fibroblasts (CAFs)

Cancer-associated fibroblasts are a major stromal component of the TME and contribute to tumor progression by remodeling the ECM, secreting pro-tumorigenic cytokines, and enhancing angiogenesis. CAFs secrete fibroblast growth factor (FGF), transforming growth factor-beta (TGF-β), and matrix metalloproteinases (MMPs), which degrade the ECM and facilitate tumor invasion and metastasis. CAFs also play a role in creating drug resistance by forming physical barriers that prevent drug penetration into the tumor 77,78.

#### 3.2.7. Extracellular Matrix (ECM)

The ECM is a structural scaffold that provides mechanical and biochemical support to cells within the TME. Tumor cells interact with the ECM via integrins and other cell surface receptors, which mediate cell adhesion, migration, and survival. Tumor-associated ECM undergoes continuous remodeling, driven by proteases such as MMPs. This remodeling creates tracks for cancer cell invasion and alters signaling pathways that promote tumor growth 79-80.

#### 3.2.8. Metabolic Reprogramming in the TME

Cancer cells in the TME often undergo metabolic reprogramming to survive in nutrient-deprived and hypoxic conditions. The reliance on glycolysis (Warburg effect) leads to lactate accumulation, acidifying the TME and suppressing immune cell activity. In addition, tumor cells compete with immune cells for nutrients like glucose and glutamine, depriving immune effector cells of the energy needed for their functions 61,34,80. Metabolic competition is a critical driver of immune evasion within the TME.

### 3.3. The Role of the TME in Cancer Progression

The TME (TME) actively participates in multiple stages of cancer progression by fostering tumor growth through the provision of essential growth factors such as EGF and VEGF, which sustain uncontrolled cellular proliferation. It facilitates immune evasion by recruiting immunosuppressive cells like Tregs and MDSCs, thereby creating a permissive environment for tumors to escape immune surveillance. Additionally, the TME promotes metastasis via CAF-mediated ECM remodeling, enabling tumor cells to invade surrounding tissues and enter the bloodstream for distant dissemination. Furthermore, hypoxia, aberrant vasculature, and ECM modifications within the TME contribute to therapy resistance by establishing physical and biochemical barriers that diminish the efficacy of chemotherapy, radiotherapy, and immunotherapy 81.

### 3.4. Targeting the TME in Cancer Therapy

The TME (TME) plays a central role in cancer development, which makes it a highly promising therapeutic target. Current therapeutic strategies for TME are focused on four key directions. In the area of immune regulation, researchers have effectively restored the body's immune surveillance function by reprogramming tumor-associated macrophages (TAMs) from a pro-tumorigenic M2 phenotype to an anti-tumorigenic M1 phenotype, while blocking the recruitment of regulatory T cells (Tregs) 69,82.

Anti-angiogenic therapy is another important strategy, and VEGF-targeted drugs represented by bevacizumab are able to normalize the disordered tumor vascular system, thus significantly improving the delivery efficiency of other therapeutic agents 83.

In terms of metabolic intervention, metabolic crosstalk between tumor cells and mesenchymal stromal cells within the TME can be effectively disrupted by strategies such as inhibiting lactate dehydrogenase A (LDHA) or targeting key enzymes of glycolysis 55,84.

Important advances have also been made in interventions targeting the extracellular matrix (ECM), which not only inhibit tumor invasion and metastasis, but also significantly enhance the tissue penetration of chemotherapeutic agents by inhibiting matrix metalloproteinase (MMP) activity or blocking ECM proteins secreted by cancer-associated fibroblasts (CAF). These multipronged therapeutic strategies provide new solution ideas to overcome TME-mediated therapeutic resistance 25,85.

### 3.5. The Role of Traditional Chinese Medicine in TME Modulation

Traditional Chinese Medicine (TCM) offers a unique advantage in targeting the TME due to its multi-target and multi-pathway mechanisms. Baicalin from Scutellaria baicalensis has been shown to inhibit IL-10 and TGF-β secretion, reprogramming TAMs from the M2 to M1 phenotype, thereby enhancing anti-tumor immunity 86. Curcumin reduces VEGF expression and stabilizes tumor vasculature, improving oxygen delivery and reducing hypoxia-induced tumor growth 87-88. Astragalus polysaccharides; an extract from Astragalus membranaceus plant enhance T cell infiltration into the TME and boost the efficacy of immune checkpoint inhibitors 89-90.

## 4. Macrophage Regulation: Detailed Insights into M1 and M2 Polarization Pathways and the Role of Traditional Chinese Medicine

### 4.1. Overview of Macrophage Polarization

**Figure 3:**
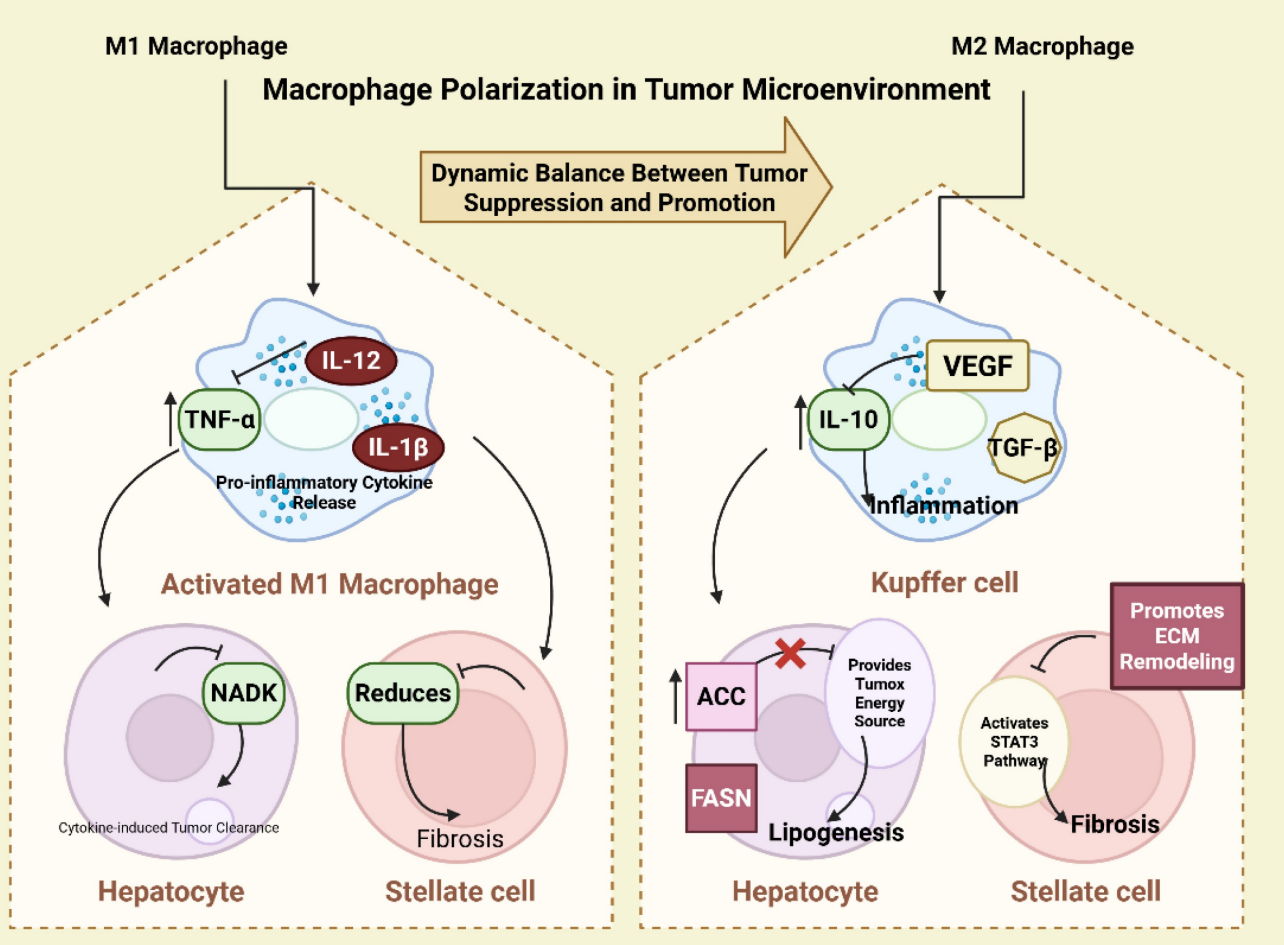
Dynamic M1/M2 macrophage polarization orchestrates TME remodeling through metabolic-immune crosstalk.

Macrophages exhibit remarkable plasticity and can adopt diverse phenotypes in response to environmental signals. Broadly, macrophages are polarized into two distinct functional states. Known as “classically activated” macrophages, M1 macrophages are induced by pro-inflammatory signals such as interferon-gamma (IFN-γ) and lipopolysaccharide (LPS). These macrophages produce pro-inflammatory cytokines such as TNF-α, IL-12, and IL-1β, and are involved in pathogen clearance and anti-tumor immunity 18,91. Referred to as “alternatively activated” macrophages, M2 macrophages are polarized by anti-inflammatory signals such as IL-4, IL-10, and TGF-β 18,92. They produce cytokines like IL-10 and TGF-β, which suppress immune responses, promote tissue remodeling, and enhance tumor progression (Figure 3).

In the TME (TME), tumor-associated macrophages (TAMs) are predominantly polarized to the M2 phenotype, which promotes angiogenesis, immune suppression, and tumor invasion. Reprogramming TAMs from the M2 to M1 phenotype is a promising therapeutic strategy to restore anti-tumor immunity.

### 4.2. Key Signaling Pathways Driving M1 and M2 Polarization

#### 4.2.1. STAT3 Pathway

**Figure 4:**
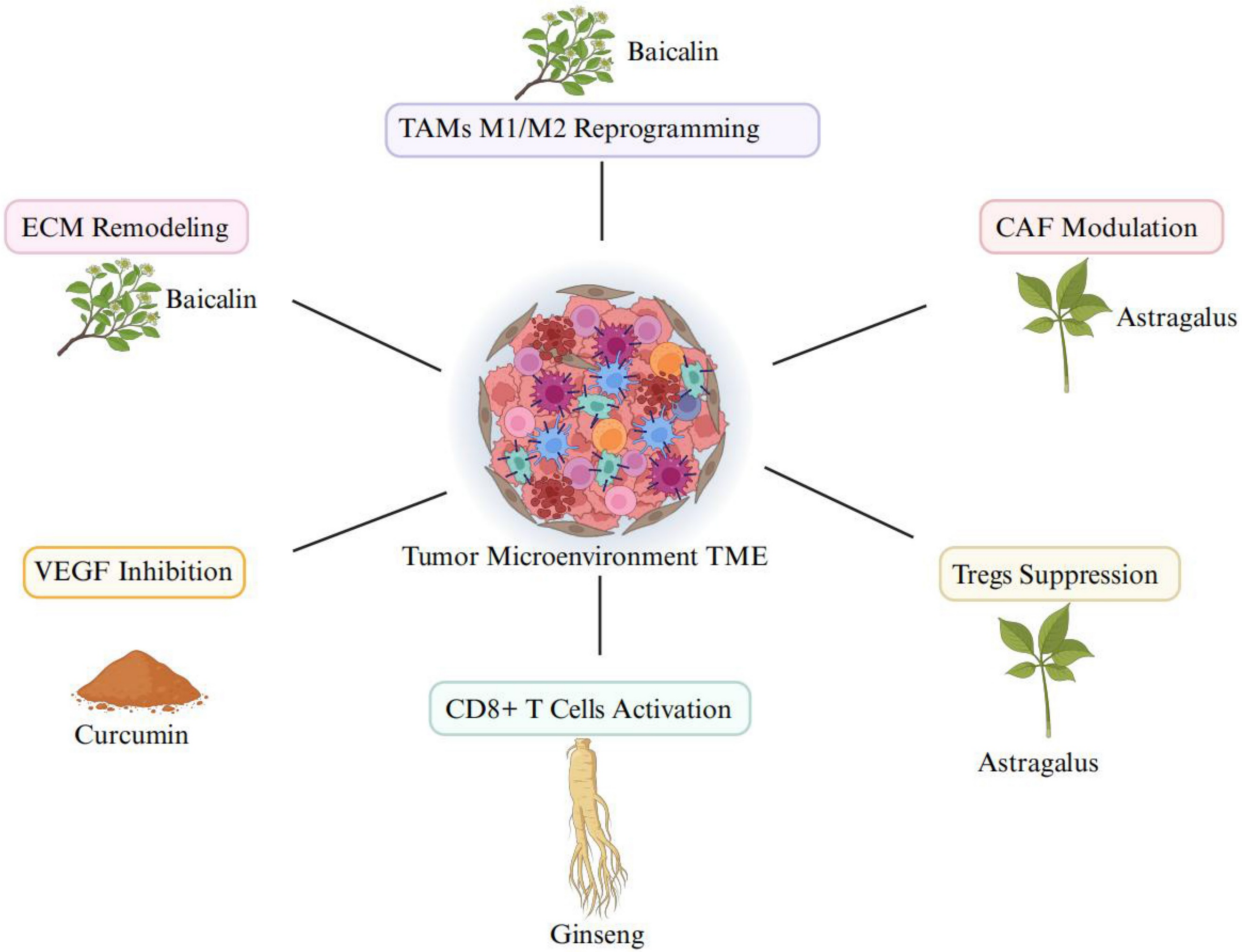
Synergistic mechanisms of multi-component traditional Chinese Medicine in remodeling the TME: triple-targeting angiogenesis, TAMs polarization, and immune ecosystem.

TCM Mechanism: *Tripterygium wilfordii* (Thunder God Vine) contains triptolide, which inhibits STAT3 activation, reducing IL-10 and Arg-1 expression in TAMs (Figure 4). The Signal Transducer and Activator of Transcription 3 (STAT3) pathway plays a pivotal role in driving M2-like polarization of tumor-associated macrophages (TAMs) within the TME. Persistent STAT3 activation, commonly induced by cytokines such as IL-6 and IL-10, promotes the transcription of immunosuppressive genes, including IL-10, VEGF, and arginase-1 (Arg-1), thereby reinforcing immune suppression and tumor progression 93.

Importantly, STAT3 signaling is closely linked to macrophage metabolic adaptation, favoring anti-inflammatory and tumor-supportive programs that stabilize the M2 phenotype 93. Inhibition of STAT3 has therefore been recognized as a critical strategy for reprogramming TAMs toward an M1-like, antitumor state.

Representative TCM components, such as triptolide derived from *Tripterygium wilfordii*, have been reported to suppress STAT3 activation, leading to reduced IL-10 and Arg-1 expression and attenuation of M2-associated immunosuppressive features 94. Collectively, these findings indicate that TCM-mediated modulation of STAT3 functions as a pathway-centered mechanism to reshape macrophage immunometabolic states, rather than isolated compound-specific effects.

#### 4.2.2. NF-κB Pathway

The nuclear factor kappa B (NF-κB) pathway is a central regulator of inflammation and macrophage polarization within the TME. Activation of the canonical NF-κB signaling cascade by pro-inflammatory stimuli such as lipopolysaccharide (LPS) and interferon-γ promotes M1-like macrophage polarization and induces the production of antitumor cytokines, including TNF-α, IL-12, and IL-1β 95.

However, under chronic inflammatory or tumor-derived signaling conditions, NF-κB activation may also contribute to immune tolerance by driving IL-10 expression and cooperating with transcriptional co-regulators that favor M2-like programs 96. Notably, NF-κB signaling is tightly linked to macrophage metabolic states, as sustained activation can support glycolysis-dependent inflammatory responses or, alternatively, immunosuppressive adaptation depending on microenvironmental cues.

Representative TCM-derived compounds, such as curcumin from *Curcuma longa*, have been shown to modulate NF-κB signaling by inhibiting IκB kinase activity and preventing NF-κB nuclear translocation, thereby attenuating M2-associated inflammatory and metabolic features while promoting antitumor immune responses 97. Collectively, these findings highlight NF-κB as a signaling hub through which TCM exerts pathway-centered regulation of macrophage immunometabolic plasticity rather than compound-specific effects.

#### 4.2.3. PI3K/AKT Pathway

The phosphatidylinositol-3-kinase/protein kinase B (PI3K/AKT) pathway is a key signaling axis governing macrophage survival, metabolism, and polarization. Activation of PI3K/AKT signaling by cytokines such as IL-4 and IL-10 promotes M2-like polarization through upregulation of immunosuppressive markers, including arginase-1 and CD206, and enhances the production of TGF-β 98,99.

From a metabolic perspective, PI3K/AKT signaling supports mitochondrial fitness and lipid metabolic reprogramming in macrophages, thereby stabilizing tumor-supportive M2 phenotypes within nutrient-rich or hypoxic tumor niches. In contrast, inhibition of PI3K/AKT signaling has been associated with a shift toward M1-like polarization and enhanced pro-inflammatory cytokine production 100.

Representative TCM interventions, such as polysaccharides derived from *Astragalus membranaceus*, have been reported to suppress AKT phosphorylation, resulting in reduced M2 polarization and increased expression of TNF-α and IL-12 in macrophages 101,102. These observations suggest that TCM-mediated modulation of PI3K/AKT signaling functions at the pathway level to reprogram macrophage immunometabolic states, rather than acting through isolated downstream targets.

#### 4.2.4. TGF-β/SMAD Pathway

The transforming growth factor-β (TGF-β)/SMAD signaling pathway is a critical driver of M2-like macrophage polarization and immunosuppressive remodeling within the TME. Elevated TGF-β levels in the TME activate SMAD2 and SMAD3, which translocate to the nucleus and induce the transcription of genes associated with immune suppression, extracellular matrix remodeling, and tumor progression 68,103. Through these effects, TGF-β/SMAD signaling not only stabilizes M2 macrophage phenotypes but also contributes to the formation of a physical and biochemical barrier that limits effective antitumor immune responses.

Representative TCM-derived compounds, such as baicalin from *Scutellaria baicalensis*, have been shown to inhibit TGF-β signaling by reducing SMAD3 phosphorylation. This modulation attenuates M2-associated immunosuppressive features and enhances T cell-mediated antitumor activity 104. Collectively, these findings suggest that TCM-mediated interference with the TGF-β/SMAD axis functions as a pathway-centered mechanism to reprogram macrophage phenotypes and reshape the immunosuppressive TME.

#### 4.2.5. Hypoxia-Inducible Factor-1 Alpha (HIF-1α)

Hypoxia is a defining feature of the TME, and hypoxia-inducible factor-1 alpha (HIF-1α) serves as a master regulator of cellular adaptation to low oxygen conditions. In macrophages, HIF-1α activation promotes M2-like polarization by upregulating pro-tumorigenic factors such as vascular endothelial growth factor (VEGF) and arginase-1 (Arg-1). In parallel, HIF-1α drives metabolic reprogramming toward glycolysis, reinforcing the functional and metabolic stability of immunosuppressive macrophage phenotypes under hypoxic stress 105.

From a therapeutic perspective, representative TCM interventions such as *Salvia miltiorrhiza* (Danshen) and its active compound tanshinone IIA have been reported to inhibit HIF-1α stabilization under hypoxic conditions. This suppression leads to reduced VEGF expression and promotes the reprogramming of tumor-associated macrophages toward a more M1-like, antitumor phenotype 106. These observations highlight HIF-1α as a key immunometabolic checkpoint through which TCM exerts pathway-level regulation of macrophage plasticity within hypoxic tumor niches. To facilitate comparison of representative traditional Chinese medicine compounds and their immunometabolic regulatory roles in macrophage polarization, a summary table is provided (Table 1).

## 5. Traditional Chinese Medicine in Metabolic Reprogramming

Cancer cells exhibit characteristic metabolic reprogramming to support their uncontrolled growth and survival. A key feature of this reprogramming is the Warburg effect, where cancer cells shift from oxidative phosphorylation (OXPHOS) to glycolysis, even under normal oxygen conditions. This shift allows for faster ATP production and provides metabolic intermediates necessary for the biosynthesis of nucleotides, lipids, and proteins, which are essential for cell division and survival. In addition to glycolysis, tumor cells also exhibit altered glutamine metabolism, lipid metabolism, and mitochondrial dysfunction, all of which contribute to the aggressive nature of tumors 27,64,107.

Traditional Chinese Medicine (TCM) compounds have shown promise in restoring metabolic balance by targeting these altered metabolic pathways. For example, Berberine, a bioactive alkaloid from Coptis chinensis, has been extensively studied for its anti-cancer effects. Berberine inhibits glycolysis by downregulating Hexokinase II (HKII), a key enzyme in the glycolytic pathway, and activating the AMP-activated protein kinase (AMPK) signaling pathway. AMPK activation leads to the suppression of mTOR, a major regulator of cell growth and metabolism, thereby reprogramming cancer cells away from glycolysis and promoting mitochondrial oxidative phosphorylation 26,42,85. Berberine also inhibits fatty acid synthase (FAS), which is crucial for lipid biosynthesis in cancer cells, and thereby reduces lipid accumulation, which is essential for tumor cell proliferation 108.

Many preclinical studies have confirmed the efficacy of curcumin, the active compound in *Curcuma longa*, in targeting cancer metabolism. Curcumin has been shown to suppress glycolysis by inhibiting Hexokinase II (HKII) and Pyruvate Kinase M2 (PKM2), both of which are overexpressed in cancer cells 109. Additionally, curcumin has been found to modulate mitochondrial dynamics, promoting mitochondrial biogenesis while inhibiting mitochondrial fission. This helps restore the normal energy balance in tumor cells, reducing their proliferative capacity. Some studies have shown that curcumin is able to affect mitochondrial function, thereby reducing the proliferation and invasion of cancer cells 28,29,110,111.

Moreover, epigallocatechin gallate (EGCG), a polyphenol derived from *Camellia sinensis* (green tea), has been shown to modulate lipid metabolism in cancer cells by inhibiting acetyl-coA carboxylase (ACC), a key enzyme in fatty acid synthesis. EGCG suppresses the mTORC1 pathway, which not only inhibits protein synthesis but also promotes autophagy, further impairing the metabolic capacity of tumor cells 28. These findings underscore the potential of TCM compounds in reprogramming cancer metabolism and restoring normal cellular energy processes.

## 6. Traditional Chinese Medicine in the TME

The TME (TME) plays a pivotal role in cancer progression by influencing the behavior of tumor cells and immune cells. One of the key features of the TME is the presence of tumor-associated macrophages (TAMs), which are crucial for promoting tumor growth, metastasis, and immune evasion. TAMs can be polarized into two distinct phenotypes: M1 macrophages, which exhibit anti-tumor activity by secreting pro-inflammatory cytokines such as TNF-α, IL-12, and IFN-γ, and M2 macrophages, which promote tumor progression and immune suppression through the secretion of immunosuppressive cytokines such as IL-10 and TGF-β 31,76. The balance between M1 and M2 macrophages in the TME is crucial in determining the overall immune landscape of the tumor.

The TME has shown considerable promise in modulating the polarization of macrophages within the TME. Baicalin, a flavonoid derived from *Scutellaria baicalensis*, has been demonstrated to promote M1 macrohage polarization while inhibiting the M2 phenotype. This shift in macrophage polarization leads to enhanced anti-tumor immunity and better tumor clearance. Baicalin exerts these effects by reducing the production of IL-10 and TGF-β, which are key cytokines involved in M2 polarization 30,88.

Similarly, Ginsenosides an active compound found in *Panax ginseng*, known for its immunomodulatory effects, enhances M1 macrophage polarization by upregulating TNF-α and IL-12 expression. Ginseng also modulates the NF-κB pathway, a critical signaling pathway in the immune response, to activate anti-tumor immune responses 33,88. Studies in mouse models have shown that Ginseng supplementation results in an increased infiltration of M1 macrophages in the TME and a concomitant reduction in tumor growth 112.

Curcumin has also been shown to modulate the TME by altering macrophage polarization. By regulating NF-κB, STAT3, and PI3K/Akt signaling pathways, curcumin promotes M1 polarization and suppresses M2 macrophage activity. This leads to increased expression of pro-inflammatory cytokines and a stronger anti-tumor immune response 113. Additionally, *Astragalus membranaceus* has been demonstrated to modulate the TME by improving the function of immune cells, including macrophages, by upregulating IL-12 and promoting T cell activation 34,36,95.

## 7. Traditional Chinese Medicine in Macrophage Regulation

Macrophages are central players in the immune system, with their ability to either promote or suppress tumor progression depending on their polarization. The balance between M1 (anti-tumor) and M2 (pro-tumor) macrophages is crucial for shaping the TME and modulating immune responses. M1 macrophages are pro-inflammatory and have anti-tumor activity, whereas M2 macrophages promote tumor progression, immune suppression, and tissue remodeling.

Studies have shown that TCM compounds have shown efficacy in modulating macrophage polarization, particularly in shifting macrophages from the M2 to the M1 phenotype. For instance, *Astragalus membranaceus*, a well-known TCM herb, has been demonstrated to promote M1 polarization by activating the PI3K/Akt pathway, which enhances pro-inflammatory cytokine production and suppresses M2 macrophage function 18,35,58. Similarly, *Tripterygium wilfordii* has been shown to inhibit the STAT3 pathway, a key regulator of M2 macrophage polarization, thereby reducing the immunosuppressive environment within the TME and enhancing the anti-tumor immune response 68.

*Polygonum cuspidatum* has been reported to inhibit M2 polarization and promote M1 polarization by modulating NF-κB signaling 114. This regulation of macrophage polarization leads to enhanced cytotoxic T cell activity and improved anti-tumor immunity.

Additionally, *Curcumin* has been shown to reduce M2 macrophage infiltration in the TME and promote the conversion of M2 macrophages to the M1 phenotype by modulating various signaling pathways, including NF-κB and STAT3. By promoting M1 macrophage polarization, TCM compounds can not only enhance anti-tumor immunity but also prevent immune evasion mechanisms commonly employed by tumors 109.

## 8. Preclinical Studies of Traditional Chinese Medicine

Preclinical studies have consistently demonstrated the potential of TCM in modulating critical pathways involved in cancer progression, particularly through metabolic reprogramming, immune regulation, and microenvironmental remodeling. A growing body of evidence indicates that TCM compounds can serve as primary agents or adjuncts to enhance the efficacy of existing cancer therapies 115.

For instance, Huangqi (*Astragalus membranaceus*), widely used for its immunomodulatory properties, has been shown to promote macrophage polarization towards the M1 phenotype in breast cancer (4T1) mouse models. This shift not only enhanced cytotoxic T cell activity but also reduced tumor growth and metastasis 35. Similarly, Triptolide, a diterpenoid derived from *Tripterygium wilfordii*, has demonstrated potent anti-cancer activity in prostate cancer models by targeting the STAT3 signaling pathway, thereby inhibiting M2 macrophage polarization and reducing the immunosuppressive environment within the TME 14,116.

In addition to immune modulation, compounds such as Baicalin have been reported to exhibit anti-metastatic properties. In preclinical models of liver cancer (HepG2), Baicalin reduced metastatic nodules in the lungs and liver by modulating the PI3K/Akt signaling pathway, which is critical for regulating macrophage polarization and tumor cell migration 30. Moreover, the potential of TCM extends beyond individual herbs. Formulae such as Shenqi Fuzheng, a combination of Astragalus and Codonopsis, have been shown to synergistically inhibit tumor growth by enhancing immune cell infiltration into the TME 117.

## 9. Mechanistic Insights of Traditional Chinese Medicine

Targeting Metabolic Pathways: TCM compounds such as berberine, curcumin, and epigallocatechin gallate (EGCG) disrupt cancer cell metabolism by modulating pathways like AMPK/mTOR and glycolysis. Berberine, for instance, activates AMPK while downregulating glycolysis-related enzymes, leading to reduced lactate production and ATP generation 26. Curcumin similarly inhibits PKM2 and mitochondrial fission, restoring oxidative metabolism in cancer cells 29.

Immune Regulation: Herbs like Astragalus and Ginseng enhance immune surveillance by promoting T cell infiltration and modulating the balance of macrophage polarization towards the M1 phenotype. This reprogramming reduces the immunosuppressive signals in the TME, such as IL-10 and TGF-β, while enhancing pro-inflammatory cytokines like TNF-α and IL-12 33,36.

Anti-Angiogenic Effects: TCM compounds like Salvianolic acid B, derived from *Salvia miltiorrhiza*, inhibit angiogenesis by suppressing VEGF expression, thereby depriving tumors of essential nutrients and oxygen. This dual effect of immune activation and vascular suppression significantly reduces tumor growth 118.

## 10. Synergistic Effects of TCM with Conventional Therapies

### 10.1. Enhancing Chemotherapy Efficacy

**Figure 5:**
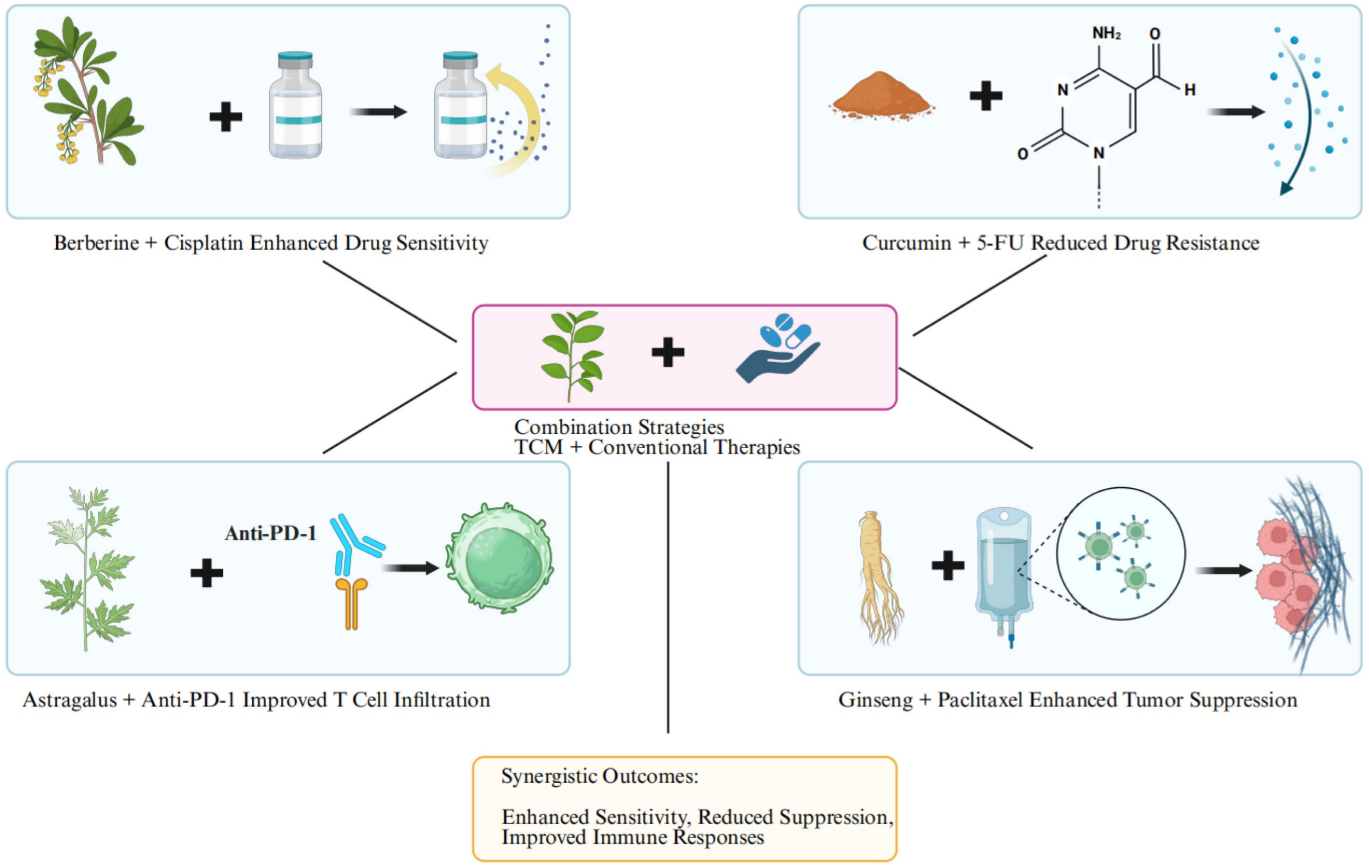
The tripartite synergy bridge of TCM-chemotherapy-immunotherapy: augmenting drug sensitivity, alleviating immune suppression, and enhancing T-cell infiltration.

The combination of TCM and chemotherapy has been extensively studied for its ability to reduce drug resistance and improve therapeutic outcomes (Figure 5). For example, berberine combined with cisplatin in ovarian cancer models demonstrated enhanced cytotoxicity against tumor cells by sensitizing them to DNA damage, while also mitigating cisplatin-induced nephrotoxicity. Mechanistically, berberine enhances cisplatin efficacy by inhibiting glycolytic pathways and activating oxidative stress responses, which synergistically promote apoptosis in cancer cells 100.

Similarly, curcumin potentiates the efficacy of 5-fluorouracil (5-FU) in colorectal cancer models by downregulating NF-κB and multidrug resistance proteins such as P-glycoprotein. This not only reduces drug resistance but also promotes cancer cell apoptosis and limits proliferation 34. Preclinical studies have also highlighted the potential of ginsenoside Rg3, a major active compound in Ginseng, which enhances the efficacy of paclitaxel in breast cancer by modulating the PI3K/Akt/mTOR pathway, thereby reducing tumor growth and preventing metastasis 42.

### 10.2. Improving Immunotherapy Outcomes

Immune checkpoint inhibitors (ICIs), such as anti-PD-1 or anti-CTLA-4 therapies, have revolutionized cancer treatment but remain ineffective in a significant subset of patients due to an immunosuppressive TME. TCM has shown promise in overcoming this challenge by modulating immune responses.

For instance, *Astragalus membranaceus* enhances the efficacy of anti-PD-1 therapy by increasing T-cell infiltration and reducing immunosuppressive cytokines like TGF-β. In a lung cancer model, combining Astragalus with anti-PD-1 therapy significantly improved tumor clearance and survival rates compared to either treatment alone 36. Similarly, curcumin has been reported to remodel the immune landscape by inhibiting M2 macrophages and enhancing cytotoxic T-cell activity, thereby improving ICI responsiveness in melanoma models 100.

## 11. Discussion

### 11.1. Challenges in TCM Research

Despite the promising preclinical and clinical data, several challenges hinder the widespread adoption of TCM in mainstream cancer therapy:

Standardization and Quality Control: The variability in TCM formulations due to differences in plant cultivation, harvesting, and preparation methods results in inconsistent therapeutic effects. Establishing standardized extraction and quality control protocols is crucial for ensuring reproducibility.

Mechanistic Complexity: TCM often exerts effects through multiple targets and pathways, making it challenging to delineate precise mechanisms of action. This complexity necessitates the use of omics technologies (e.g., transcriptomics, proteomics, and metabolomics) to comprehensively evaluate its biological effects.

Regulatory Barriers: Many TCM compounds lack rigorous clinical validation, limiting their acceptance by regulatory bodies. Conducting large-scale, randomized controlled trials (RCTs) with standardized protocols is essential to overcome these barriers. Although limitations such as formulation standardization and variability in clinical evidence have been acknowledged, future efforts should further address these challenges through well-designed multicenter, large-sample clinical trials. Such studies are essential to enhance reproducibility, reduce center-specific bias, and provide robust evidence for the clinical efficacy and safety of traditional Chinese medicine (TCM) in oncology. In addition, future research should focus on the rational integration of TCM into existing cancer treatment paradigms. Rather than serving as a stand-alone intervention, TCM may function as an adjuvant modality that complements immunotherapy and targeted therapy by modulating the TME, alleviating treatment-related immunosuppression, and improving therapeutic responsiveness. Exploring combined treatment strategies in different tumor contexts may therefore facilitate the clinical translation of TCM and optimize its role in personalized cancer management.

### 11.2. Future Directions in TCM Research

Integration of Advanced Technologies: The use of molecular docking, high-throughput screening, and AI-based prediction models can accelerate the discovery of bioactive compounds in TCM and their molecular targets. For example, AI can be used to predict synergistic combinations of TCM and conventional therapies.

Development of Combination Therapies: Synergizing TCM with modern cancer therapies such as immunotherapy and targeted therapies, and exploring the potential of combining TCM with emerging therapies such as CAR-T cells, oncolytic viruses, or nano-drug delivery systems may open new avenues for cancer treatment.

Personalized Medicine: Leveraging precision medicine approaches to tailor TCM treatments based on individual genetic and metabolic profiles could enhance efficacy and minimize side effects.

Global Collaboration: Encouraging international collaboration in TCM research, particularly with institutions specializing in cancer biology and pharmacology, can facilitate cross-disciplinary studies and clinical applications.

Clinical translation: Validation of the effects of TCM on metabolic pathways in human trials, e.g., designing phased clinical trials to objectively assess the effects of TCM interventions on metabolic reprogramming.

Bioavailability issues: The low bioavailability of many TCM compounds is addressed using nanotechnology or improved delivery systems.

Despite encouraging preclinical evidence, the clinical translation of TCM in oncology remains constrained by several challenges. These include heterogeneity in patient populations, variability in TMEal states, and the lack of standardized clinical endpoints that directly reflect immunometabolic modulation. To overcome these limitations, future studies should prioritize biomarker-guided clinical trial designs, integrate immune and metabolic profiling for patient stratification, and adopt multicenter, adequately powered trial frameworks. Importantly, TCM is more likely to exert clinical benefit as an adjuvant rather than a stand-alone therapy. Given the immunometabolic heterogeneity across tumor types, the integration of TCM with conventional treatments should therefore be context-dependent. Tumors characterized by hypoxia, lactate accumulation, and M2-dominant macrophage infiltration may benefit from TCM interventions targeting metabolic normalization and macrophage reprogramming to enhance responsiveness to immunotherapy. In contrast, tumors with pre-existing immune infiltration but pronounced therapy-induced immunosuppression may derive greater benefit from TCM-based adjuncts during chemotherapy. Together, these considerations highlight the potential of TCM as a flexible adjuvant modality that complements established anticancer therapies by reshaping the TME rather than replacing standard treatments.

## Figures and Tables

**Table 1:** Immunometabolic regulatory roles of representative TCM compounds in tumor-associated macrophages.

TCM compound / herb	Primary signaling pathway	Metabolic regulation	Immune modulation (TAMs)	Key functional outcome
Triptolide (Tripterygium wilfordii)	STAT3	Suppression of M2-associated metabolic programs	↓ IL-10, ↓ Arg-1, reduced M2 polarization	Restoration of antitumor immune activity
Curcumin (Curcuma longa)	NF-κB	Modulation of inflammatory metabolism	Shift from M2 to M1 phenotype	Enhanced pro-inflammatory immune response
Astragalus polysaccharides (Astragalus membranaceus)	PI3K/AKT	Regulation of lipid and mitochondrial metabolism	↓ M2 markers, ↑ TNF-α / IL-12	Promotion of M1-like macrophage polarization
Baicalin (Scutellaria baicalensis)	TGF-β/SMAD	Attenuation of immunosuppressive signaling	Inhibition of M2 polarization	Enhanced T cell-mediated antitumor immunity
Tanshinone IIA (Salvia miltiorrhiza)	HIF-1α	Reduction of glycolysis under hypoxia	Reprogramming of TAMs toward M1	Alleviation of hypoxia-driven immune suppression
